# Application of the Anammox in China—A Review

**DOI:** 10.3390/ijerph17031090

**Published:** 2020-02-09

**Authors:** Ruolan Wen, Yue Jin, Wenjie Zhang

**Affiliations:** 1 Guangxi Key Laboratory of Theory & Technology for Environmental Pollution Control, College of Environmental Science and Engineering, Guilin University of Technology, Guilin 541004, China; wrl20190908@163.com (R.W.); 2010053@glut.edu.cn (W.Z.); 2 Guangxi Collaborative Innovation Center for Water Pollution Control and Water Safety in Karst Area, Guilin University of Technology, Guilin 541004, China; 3 College of Civil Engineering and Architecture, Guilin University of Technology, Guilin 541004, China

**Keywords:** anaerobic ammonia oxidation, nitrite, sequencing batch reactor, engineering application

## Abstract

Anaerobic ammonia oxidation (anammox) has been one of the most innovative discoveries for the treatment of wastewater with high ammonia nitrogen concentrations. The process has significant advantages for energy saving and sludge reduction, also capital costs and greenhouse gases emissions are reduced. Recently, the use of anammox has rapidly become mainstream in China. This study reviews the engineering applications of the anammox process in China, including various anammox-based technologies, selection of anammox reactors and attempts to apply them to different wastewater treatment plants. This review discusses the control and implementation of stable reactor operation and analyzes challenges facing mainstream anammox applications. Finally, a unique and novel perspective on the development and application of anammox in China is presented.

## 1. Introduction

With the rapid development of urbanization and industrialization in China, the discharge standards of sewage have become increasingly strict and will become progressively more difficult to meet in the future. Not only are treatment costs increasing but the removal of nitrogen particularly total nitrogen, is becoming less effective. In 2012, the average ammonia nitrogen removal rate of treated wastewater was only 37% [1]. Moreover, several limitations of the traditional nitrification and denitrification process have gradually emerged, such as high energy consumption, high operation costs, low-level ammonia nitrogen removal and unsatisfactory disposal of excess sludge. Table 1 [2] compared the differences between traditional nitrification-denitrification process and anaerobic ammonia oxidation (anammox) process.

The anammox process was discovered in the 1990s, whereby, ammonia nitrogen and nitrite nitrogen are removed simultaneously with the generation of N_2_ [3]. Compared to traditional nitrification and denitrification process, the anammox process uses nitrite as an electron acceptor, without the direct ammoxidation of O_2_ into N_2_. This unique method of oxidation in wastewater treatment has obvious advantages, for example oxygen demand is reduced by 60%, organic carbon demand is reduced by 100% and the sludge yield is reduced by 90% [4,5,6,7]. Nearly energy-neutral or even energy-positive treatment schemes are possible, when anammox technology is included. Therefore, it has become a hot spot of water treatment research.

At present, anammox technology is predominantly still at the laboratory stage in China and most of the experimental water is artificial water. Moreover, anaerobic ammonia oxidation bacteria (AnAOB)—a specific anaerobic bacteria for chemical energy autotrophy—has a slow growth rate with a doubling time of 10–30 days [8], strict requirements for the growth environment and a difficult start-up progress, which are the most significant obstructions to practical engineering application of the anammox process [9].

Each technology has its own characteristics and adaptability. Despite the huge advantages of the mainstream anammox process, it is important to fully assess its current applicability in China. From the perspective of general control methods and strict effluent quality, further work is required before anammox can be applied as a mainstream process. Moreover, it is not expected that anammox will immediately replace traditional nitrification and denitrification process but will instead be employed as a temporary side flow process. It is believed that continued in-depth exploration and research in this field will provide a more theoretical basis for future engineering applications of anammox [9].

This study reviews research and development related to the anammox process, including the various achievements and obstacles to its comprehensive application in China. Then, the characteristics and properties of different anammox processes are analyzed. The experience in operation and the challenges in the process of technology expansion are summarized. At present, anammox has been successfully applied to the treatment of monosodium glutamate wastewater, pharmaceutical wastewater, manure wastewater and landfill leachate. At the same time, the development of anammox in the world and China is analyzed and compared. According to the current situation of wastewater treatment in China, the future development of anammox is proposed and the potential application market of anammox in China is prospected.

## 2. Review of Anammox Engineering Projects

In order to remove ammonia nitrogen from wastewater through the anammox process, it is necessary to provide enough nitrite as the electron acceptor [10,11]. However, there is almost no nitrite in typical wastewater [12,13]. Therefore, ammonia must be partially transformed from aerobic autotrophic ammonia oxidizing bacteria (AOB) to nitrite, before AnAOB can convert the generated nitrite to N_2_ [14]. By integrating shortcut nitrification and anaerobic ammoxidation (PN/A), a variety of processes have been developed, such as SHARON-Anammox, OLAND, CANON and deammonification process (DEMON) (Table 2).

**Figure 1:**
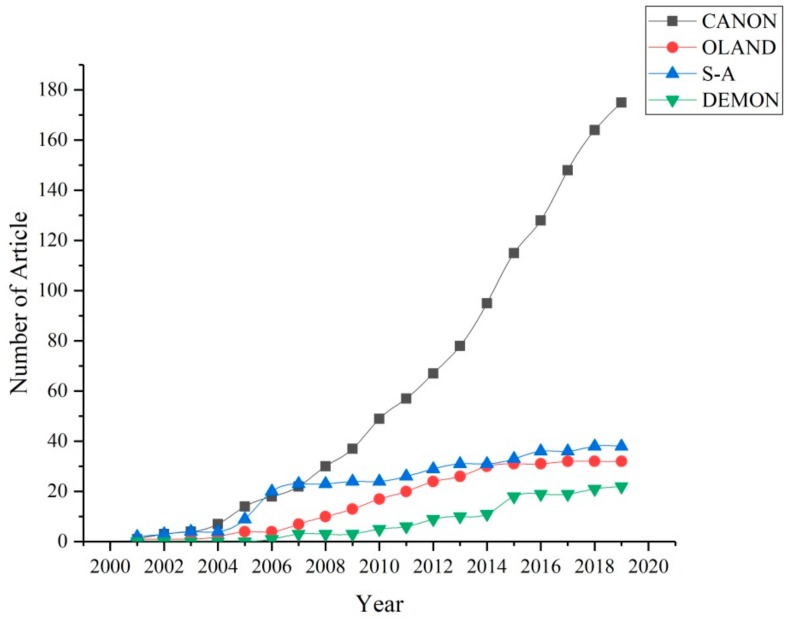
Common anammox process types (Data source: Web of Science).

SHARON-Anammox, a secondary treatment process, was developed in the early stage of anammox development [7]. However, since 2008, the number of relevant studies has decreased significantly. The research focus has gradually turned to CANON and to a lesser extent OLAND and other primary treatment processes (Figure 1), indicating that the primary process will become an increasingly popular research prospect topic in the future.

**Figure 2:**
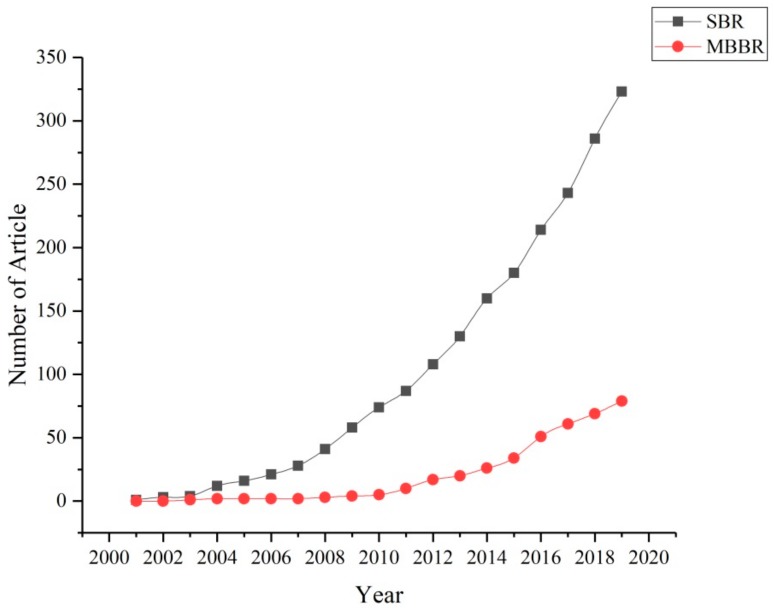
Previous studies about sequencing batch reactor (SBR) and moving bed biofilm reactor (MBBR) for the anammox process (Data source: Web of Science).

Due to the slow growth rate of AnAOB, an anammox reactor must have efficient biomass retention capacity; therefore, the reactor was studied. At present, moving bed biofilm reactors (MBBRs), granular sludge systems and SBRs are widely used. As shown in Figure 2, SBR is the most popular reactor, followed by granular sludge systems and MBBR. Anammox in different reactors is discussed below:

### 2.1. SBR

The most widely used process in an SBR is the DEMON process [19], which is characterized by controlling pH value and using a hydro cyclone to separate anammox bacteria. By adjusting the sludge retention time of AOB and AnAOB, slowly growing AnAOB bacteria are isolated to maintain the anaerobic flora [20]. Due to the high concentration of ammonia nitrogen in the anaerobic sludge digestion liquid and sludge dewatering liquid, the flow measurement anammox demo process was adopted in the Strass wastewater treatment plant in Austria [21]. By 2014, there are more than 30 sewage plants using DEMON technology around the world [22].

Another widely studied process, CANON, was developed in 2001 [23] and is also known as a fully autotrophic denitrification process. For example, CANON is adopted in the Meihua Industrial Park of Tongliao, China [16]. The CANON reactor is fully automatic and can continuously monitor DO and pH value online and record the data in electronic form. During operation of the CANON process, air and nitrogen are automatically and alternately supplied [24]. In the aerobic stage, the stirring speed is controlled at a higher level, whereas in the anaerobic stage, the stirring speed is controlled at a lower level.

The OLAND process requires strict control of aeration [25]. The mechanism is similar to that of CANON, as shown in Table 3 [26]. At present, the OLAND process predominantly adopts the RBC reaction system, which is rare in practical engineering but expected to be more widely applied in future

### 2.2. Biofilm Reactor

In the biofilm reactor, oxygen can be consumed by the AOB in the outer layer, whereas AnAOB can grow in the anoxic inner region; therefore the biofilm reactor is very suitable for the anammox process [27]. MBBR is currently the dominant biofilm treatment process in the Hagentin sewage treatment plant in Germany. MBBRs are equipped with 40%–50% carrier, an agitator and aeration [28,29].

With the development of MBBR research, a new process, single-stage deammonification process utilizing biofilms on moving carriers in a mixed reactor (ANITA Mox) [20], has been adopted in Malmö, Sweden. The patent method was used to control aeration, whereas the ratio of ammonium concentration, both in and out of water and nitrate production were used to control DO. Another process, integrated fixed membrane activated sludge (IFAS), adds a settler to the system, which improves the performance by 3–4 times [20]. The suspended sludge from the effluent accounts for 90% of the total aerobic AOB and has a higher biological turnover rate (sludge output) than pure biofilm [30].

Azari et al. [31] used a combination of the activated sludge method and an activated carbon biofilm reactor to treat the leachate of the municipal waste treatment plant in Herten, Germany. The average total nitrogen removal rate from wastewater was 94% but 82 ± 6% without the activated carbon reactor. Moreover, the consumption of energy, methanol and excess sludge was significantly reduced. Thus, the combination of activated carbon biofilm and activated sludge can achieve better oxidation of ammonia.

### 2.3. Granular Sludge

Granular sludge has the same function as biofilm. The outer aerobic environment is suitable for AOB growth, whereas the inner anoxic environment is suitable for AnAOB growth. Therefore, no additional carrier materials are required in the granular sludge system. By 2007, the first full-scale granular anammox reactor was implemented at the sewage treatment plant in Rotterdam in the Netherlands [7]. SHARON process is used for removal of ammonium nitrogen from wastewater by partial nitrification of ammonium nitrogen to nitrites at 25–30 °C. It was originally designed for reducing oxygen and organic carbon supply for the traditional denitrification process. SHARON process provides nitrite for anammox process; therefore, SHARON process can be coupled with anammox as two stage process. 

In recent years, anammox particles have been comprehensively studied in various bioreactors; however the mechanism of granulation is rarely studied [32]. Additionally, particle flotation is an important factor leading to instability of the anaerobic reactor. In the face of these problems, some research groups in China [33,34] have found ways to overcome floating particles and restore reactor performance. The floating particles are removed from the reactor, broken into small pieces and returned to the reactor to solve the problem of anammox sludge floating.

### 2.4. New Technologies

With ongoing research, additional new technologies have been applied to the anammox process. Kazuichi et al. [35] used gel encapsulation technology to treat ammonia containing wastewater with a nitrogen concentration of 690 mg/L and a design load of 400 kg-N/d. Their study showed that, although methanol is contained in the wastewater, it can be removed during pretreatment. This technology makes the PN/A start up faster and exhibits stable nitrification performance. Therefore, the application of this technology can benefit the further application of anammox to the treatment of industrial wastewater.

### 2.5. Engineering Projects in China

**Figure 3:**
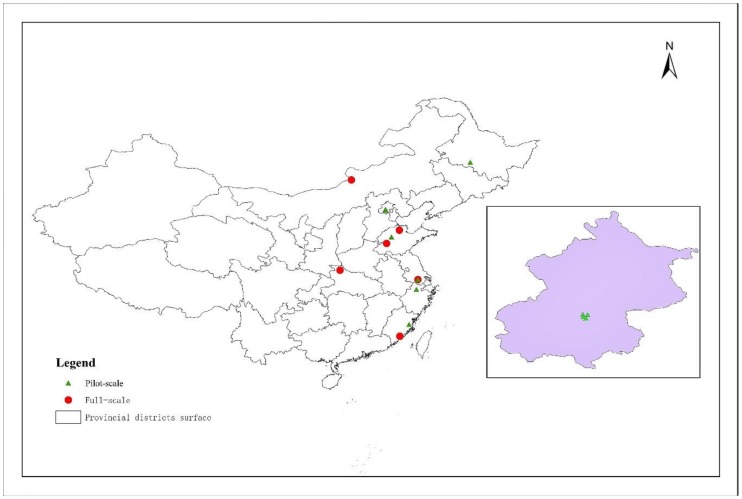
Geographical distribution of pilot-scale and full-scale anammox plants in China.

In 2012, there were 3340 sewage treatment plants in China, with a sewage treatment capacity of approximately 1.42 × 10^8^ m^3^/d [36]. Furthermore, the number of sewage plants employing anammox bacteria is also increasing. Chinese research into the engineering applications of anammox has typically remained at the pilot test stage and small industrial scale stage, in which the operating volume of the reactor is between 0.020 m^3^ and 22.5 m^3^ and an upflow anaerobic sludge blanket (UASB) reactor is used to treat different types of wastewater [32]. An et al. [37] successfully operated a pilot-scale anammox reactor to treat dry spinning acrylic fiber wastewater; by controlling the reactor temperature and influent suspended solids, the removal rate of ammonia nitrogen can be improved. Thus, operation of a pilot-scale reactor is crucial for the future application of anammox. Figure 3 describes the distribution of pilot-scale and comprehensive application scale anammox in China. It can be seen from the figure that the pilot-scale experiment of anammox is mainly carried out around research centers and universities and has the characteristics of rapid development in coastal areas.

With continued developments in research, UASB reactors are not only used in the laboratory. The Hubei Shiyan landfill leachate treatment plant adopts a combined two-stage UASB, anammox and membrane bioreactor and reverse osmosis (MBR/RO) treatment process. The designed daily treatment capacity is 150 m^3^, the COD effluent is controlled at 100 mg/L, the total nitrogen effluent is controlled at 40 mg/L and the ammonia nitrogen effluent is controlled at 25 mg/L. This process is the first project in China to use anammox to treat landfill leachate, as well as to solve the low carbon nitrogen ratio of landfill leachate. The successful operation of the Hubei Shiyan landfill leachate treatment project provides a case study of anammox technology in China achieving high efficiency, energy savings and reduced consumption.

After anaerobic and aerobic treatment of industrial wastewater from the Angel Yeast company in Binzhou, Shandong Province, the concentration of ammonia nitrogen in the effluent was approximately 300–800 mg/L, which not only does not meet the discharge standard but also has a harmful impact on the surrounding residents. Therefore, the company introduced the ANAMMOX^®^ process technology from the Paques company to treat high ammonia nitrogen wastewater, representing the first practical application of ANAMMOX^®^ technology in the field of yeast wastewater treatment. The 500 m^3^ ANAMMOX^®^ reactor replaces the 10,000 m^3^ anoxic oxic (A/O) process. The bioreactor operates stably under an ammonia nitrogen load of 2 kg-N/m^3^/d; this is currently the highest sludge load the ANAMMOX^®^ reactor can carry on an industrial scale, which is far higher than that under the traditional process.

Tongliao Meihua Industrial Park adopts a single-stage anammox reactor with a design capacity of 11,000 kg-N/d to treat wastewater from monosodium glutamate production [38]. The park is one of the largest sewage treatment plants in China; 10 times larger than the largest sewage treatment plant in China prior to 2008. Shandong Xiangrui Pharmaceutical Co., Ltd. employs a 4300 m^3^ anammox reactor to treat corn starch and monosodium glutamate production-related wastewater. The designed ammonia nitrogen load is 1.42 kg-N/m^3^/d. The successful implementation of these anammox projects will greatly accelerate the application of anammox as the main wastewater treatment process in China. 

**Figure 4:**
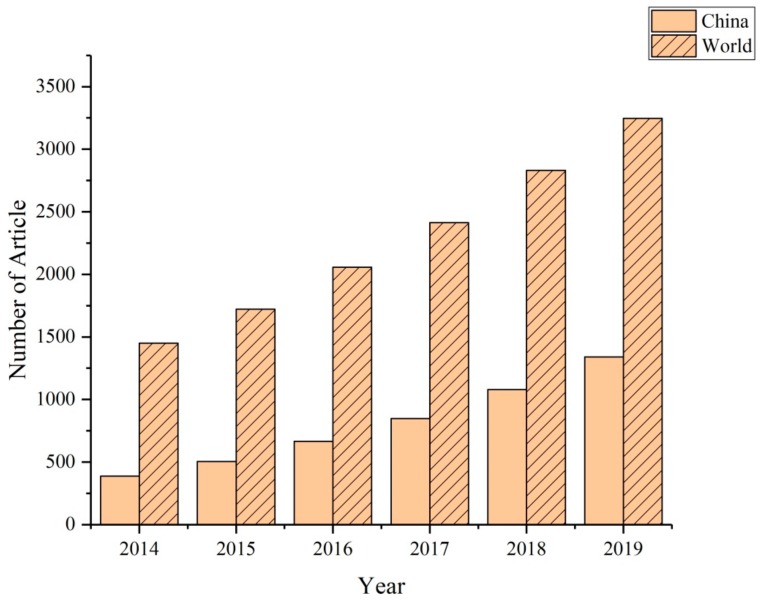
Number of anammox-related studies published in China and around the world (Data source: Web of Science).

The design and practical implementation of anammox in China predominantly implemented by the Paques company of the Netherlands [32]. Until 2013, five of the 11 anammox wastewater treatment plants constructed by the Paques company have been built in China. As the largest developing market in the world, China has made significant contributions not only to the commercialization and engineering of anammox but also to relevant theoretical research (Figure 4).

In addition to the introduction of foreign technology, some Chinese research teams have used their own expertise and technology to apply the anammox process to the field of engineering. The research group of Yan’an Biotechnology Co., Ltd. of Zhejiang University has successfully implemented pilot-scale anammox sewage treatment plants in Yiwu City, treating monosodium glutamate wastewater (60 m^3^) and pharmaceutical wastewater (10 m^3^), respectively [32]. Research into anammox wastewater treatment plants has gradually moved from the laboratory to full-scale wastewater treatment plants. The application of anammox technology to different types of wastewater are listed in Table 4.

## 3. Problems and Solutions for Anammox Engineering Applications

Since the mainstream anammox process was proposed in 1990, the PN/A process model has been widely used around the world [46]. However, due to the complex bacterial flora in the reactor, the operational process is not always in an ideal control state [10]. The first challenge is the high carbon to nitrogen (C/N) ratio of municipal wastewater after one-time sedimentation [47]. In addition, nitrite oxidizing bacteria (NOB) can be effectively inhibited during the flow measurement process, where the concentration of free ammonium and free nitrous acid is higher than the threshold value; however, due to the lower concentration of ammonium nitrogen in the mainstream process, the inhibition of NOB becomes another major challenge to its mainstream application [48]. In addition, the activity of AnAOB is reduced in low temperature when urban wastewater exhibiting seasonal temperature changes. Because of complex bacterial flora in the reactor and the need to control the appropriate reaction conditions, a strong online control system and more reliable monitoring parameters are required.

### 3.1. On-Line Monitoring

To ensure the robust practical application of anammox, on-line monitoring is indispensable during operation, which ensures for the stable and safe operation of the anammox reactor. Online monitoring items typically include DO, pH, ammonia, nitrate and nitrite; however, the complex reaction process of PN/A, leads to higher requirements for online sensors. Although a large number of sensors are used in the monitoring process, most online monitoring is still only based on pH and DO concentration [10]. Due to the unstable correlation between the bacteria and their concentration in the reactor, most of the parameters are related to DO concentration, errors may occur if only DO is measured [14,49,50]. However, monitoring the air flow rate as well as the type of nitrogen can provide a more accurate basis for the stable operation of the reactor [51].

In addition to DO and pH monitoring, Langone et al. [52] monitored an SBR reactor and found that the characteristic curve of conductivity was closely related to the biological reaction process. Moreover, the distribution of conductivity was directly related to the reduction in ammonium concentration.

Zekker et al. [53] used an oxidation–reduction potential (ORP) decrease rate control as a novel anammox treatment step, shortening the control parameter, which was gradually decreased at values of 1.65, 0.9 and 0.4 mV/min, ensuring high total NRR and low accumulation of ammonium and nitrate.

Monitoring the air flow rate, conductivity and ORP can provide better reference data for the stable operation of anammox.

### 3.2. Regulation of Functional Bacteria

**Figure 5:**
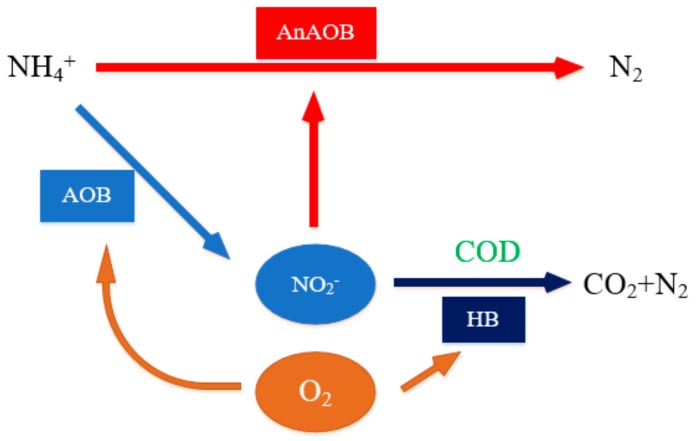
Competition among ammonia oxidizing bacteria (AOB), anaerobic AOB (AnAOB) and heterotrophic bacteria (HB).

Operation in PN/A mode is based on the coexistence and effective cooperation between AOB and AnAOB [54]. Maintaining biomass is an important design parameter of the anammox operation; therefore, in engineering applications, it is crucial to ensure that AnAOB is the dominant strain [55]. In the mainstream process, in addition to the need to coordinate growth between AOB and AnAOB, the control of heterotrophic bacteria (HB) is a decisive link in the anammox design. A high C/N ratio of influent water will stimulate the growth of HB on the surface of particles or biofilm, affect the mass transfer, limit the acquisition of oxygen by AOB and change the biofilm stratification [56]. Figure 5 reflects the competition between HB, AOB and AnAOB in the presence of COD which further illustrates that the C/N ratio of influent water is an important parameter for controlling the mainstream process [20].

NOB control is a major challenge for the mainstream PN/A process. The growth rate of AOB is almost the same as that of NOB in the approximate temperature range of 10–20 °C, which makes it impossible to screen NOB effectively [57,58]. At present, the strategy of NOB inhibition includes maintaining the concentration of ammonia nitrogen in the influent at more than 2 mg/L (the growth rate of NOB is faster than that of AOB at low substrate concentration), instantaneous anoxia (real-time aeration control), which is proved operational feasible in the Strass sewage treatment plant [59]. The latter is achieved by setting the DO or flow rate to inhibit NOB, however the reliability of key equipment such as the blower is crucial to the stable operation of the process [60,61].

### 3.3. Substrate

In most sewage treatment plants, ammonium is one of the main forms of nitrogen [62]. However, the process of anammox is less affected by the concentration of ammonium, than by the amount of free ammonia (FA); therefore, the key inhibitor of ammonium is FA concentration. Fernández et al. [63] found that the performance of anammox sludge became unstable when the FA concentration was greater than 35–40 mg/L for a long time. Thus, maintaining the FA concentration below 20–25 mg/L can ensure the stable operation of anammox.

The presence of nitrite ensures anaerobic ammoxidation; however, when the concentration of nitrite is too high, the reaction is inhibited [26]. However, because experiments are typically conducted under different operating conditions, the inhibition threshold, inhibition degree, reversibility and even location vary substantially; therefore, the results cannot easily be applied to practical operations. For example, Kimura et al. [64] encapsulated anammox bacteria in a gel carrier to study the effect of nitrite on anammox bacteria. Their study showed that anammox bacteria can be greatly enhanced by incorporation into the gel carrier; however, this also leads to an increase in the mass transfer resistance and a reduction of denitrification efficiency.

Although nitrate accumulation is not a key inhibiting factor, nitrate accumulation will partially affect the microbial community balance in the reactor [51]. The main reason for the accumulation of both nitrite and nitrate is that the reactor aeration is too high [59]. In engineering, the air flow and blower power or operation time can be reduced and in an SBR, a hydro cyclone can also be used to remove flocculated sludge and control the accumulation of nitrate [26].

In the anammox projects, the concentration of FA, nitrite and nitrate should be strictly monitored, particularly that of nitrite during the start-up period of the single-stage reactor, when the growth rate of AOB is faster than that of AnAOB, higher nitrite concentration may have an impact on the anaerobic flora [65]. In view of the above problems, aeration can be reduced or stopped completely depending on the situation in order to reduce the water inflow load and biomass.

### 3.4. DO and Total Suspended Solids (TSS)

DO concentration is the main control parameter of sewage treatment [66]. During anammox system operation, excessive DO will lead to the growth of NOB, compete with AnAOB and AOB for nitrite and oxygen and cause the accumulation of nitrogen [66,67]. Failure to monitor DO signal will have serious consequences for the operation process; thus, Joss et al. [51] proposed a method for measuring air flow instead of DO concentration that will provide better and more reliable control parameters.

The other factor with the greatest impact on the PN/A process is the solid concentration in the influent water. When the TSS of influent water is too high, the output of nitrate will increase [10]. Additional sludge needs to be discharged to increase sludge loss, which also reduces the biomass in the reactor, resulting in a reduction of activity. Therefore, such pretreatment should be considered when the TSS of the influent water is high.

### 3.5. pH and Temperature

Hydrogen ions (a common pH indicator) are consumed in the anammox process; therefore, the pH signal can represent the current state of the reaction and indicate the actual duration of the aeration interval during operation. The efficiency of the anammox process will be greatly reduced when pH values are outside of optimum range for the autotrophic bacteria. Thus pH should be regulated within the foreseeable fluctuation range.

In engineering, temperature is an important factor but difficult to adjust. For example, Changi sewage plant in Singapore is the first sewage treatment plant to achieve stable mainstream anammox operation [56], which is inextricably linked to the high water temperature in Singapore (28–30 °C).

Durán et al. [68] showed the potential for partial nitrification at low temperature by controlling the aeration frequency, pH value [69] and DO concentration [70] or by adding hydroxylamine to inhibit the activity of NOB.

Bowen et al. [71] showed that a granular anammox reactor can achieve stable operation at low temperatures (down to 15 °C) when treating low strength synthetic wastewater using intermittent high-concentration influent and gradual cooling. After three weeks of domestication at 15 °C, the NLR was 1.23 kg-N/m^3^/d and the NRR was 0.82 ± 0.03 kg-N/m^3^/d. Over long-term operation, NOB was successfully inhibited and showed no adaptability in the low FA environment. Li et al. [72] studied a new method to enhance the reactor performance enriched with marine anammox bacteria (MAB) by using Fe (III). This enables SBRs to operate at low temperatures (15 °C). Therefore, recent experimental progress has proved the feasibility of the mainstream PN/A process at low temperature; however, more research and experiments are required.

### 3.6. Heavy Metals and Antibiotics

In addition to the inhibition of substrate concentration on anammox process, wastewater also contains heavy metals such as copper, zinc, nickel, lead and antibiotics, for example, municipal landfill leachates, piggery wastewater and semiconductor manufacturing wastewater streams [73]. Yang [74] and Achlesh [75] studied the activity of Cu (II) and Zn (II) on Anammox, respectively. However, heavy metals usually exist simultaneously in wastewater, so the research direction should focus on the joint effect of heavy metals on the anammox process [76].

Lotti et al. [77] found that, after 24 hours of exposure, the IC_50_ of copper and zinc were 1.9 and 3.9 mg/L, respectively. For copper, especially at concentrations higher than 2 mg-Cu/L, there seemed to be a clear exposure time effect (activity declined with increasing exposure time) while for zinc the effect is not obvious. Zhang et al. [78] studied the inhibition of anammox in the presence of divalent copper and zinc. With the increasing concentration, the inhibition increased first and then weakened. The strongest inhibition concentrations of copper and zinc were 16.3 and 20.0 mg/L, respectively. In addition, in the absence of substrate, copper strongly inhibited the activity of Anammox, while the presence of nitrite significantly enhanced the inhibition

Antibiotics in wastewater are usually difficult to remove. Oxytetracycline (OTC) is an antibiotic widely used in animal husbandry, which is common in manure wastewater. Shi et al. [79] studied the effect of OTC on the activity of anammox sludge. When exposed to 2 mg/L of OTC, the denitrification capacity of the anammox reactor was almost completely lost within three weeks and the specific activity of the AnAOB was also significantly reduced. When the influent does not contain OTC, the denitrification capacity of the anammox reactor is restored. Fernández et al. [80] found that tetracycline hydrochloride had a strong inhibitory effect on the anammox process in the concentration range of 100–000 mg/L and chloramphenicol in the concentration range of 250–1000 mg/L. The presence of heavy metals and antibiotics in wastewater can inhibit the activity of anammox in varying degrees. Therefore, the pretreatment of heavy metals and antibiotics should be carried out to avoid affecting the operation of subsequent processes.

## 4. Discussion

There are various problems in the operation process, future research should be carried out to overcome the obstacles revealed in this paper. First the optimizing operation conditions are suggested to be focused on. At present, monitoring the air flow rate, conductivity and ORP has been successfully applied, in addition, it should be specially developed based on an automatic process control system. Secondly, little is known about the effect of some influent components on the reaction process; thus, research into “inhibition” will still be a hot topic in the future. Although different types of wastewater have been proven suitable for anammox under laboratory conditions, the complex composition of different wastewater generates substantial challenges for the engineering application of the anammox process. Then, we should also pay attention to the interaction of key microbiota, to clarify the entire complexity of the mainstream anammox technology. In this paper, the negative effects of heavy metals and antibiotics on anammox were introduced. There is no specific solution for this kind of influence, which should be based on prevention and avoided in the main reactor as much as possible. Moreover inorganic salts, sulfides, phenols and other components, the compatibility of which has not been fully studied [81,82,83]. In addition, the negative effect of N_2_O on the environment should be considered [84,85,86].

Furthermore, with the development of research, the problem of low temperature operation has been solved gradually but lower ammonia concentrations and higher denitrification requirements are the main challenges faced by the mainstream anammox process. Therefore, it is necessary to develop effective ammonia removal methods for mainstream urban sewage treatment and methods for achieving bacterial amplification at low substrate concentration and temperature [87,88].

In addition, the engineering application of anammox should not only focus on the research of new reactors but also consider the feasibility of technology replacement of anammox in the existing sewage treatment plants. The reinforced concrete equipment such as the reaction tank of the sewage plant will not be changed easily and can be used for more than 100 years. Therefore, the combination of new technology and such equipment should be considered. The A/O process is one of the common sewage treatment processes, which can be used as the pretreatment of PN/A process. SBR reactor is very popular in the application of anammox. Among the built sewage treatment plants in China, the most popular one is oxidation ditch, the second is A/O process and the third is SBR [36].

## 5. Conclusions

In recent years, China has made increasingly large investments in anammox research. Particularly pertinent issues related to this process as we look forward include: (1) Researchers pay more and more attention to reactors, microbial action, wastewater type and process type. The control difficulties in the operation process, such as aeration, temperature, nitrite accumulation, are gradually solved. (2) According to statistics of published research, China has published the most articles on anammox (220, 27.71%), followed by the Netherlands (189, 23.8%) and the United States (159, 20.03%) [89]. Although it is not widely in full-scale, adequate theories lay a foundation for subsequent applications. (3) With the construction of China’s first urban sewage concept plant in Yixing in May 2018, this will become the first sewage treatment plant to adopt the “carbon and phosphorus efficient separation + mainstream anammox + fine treatment” process. Facing the huge sewage treatment market in China and huge increase in research attention, China is expected to become a significant market for anammox in the near future.

## Figures and Tables

**Table 1:** Anammox or its combinations compared to traditional nitrification-denitrification.

Processes	Microorganisms	NLR kg-N/m^3^/d	Biomass Production Rate kg/kg-N	DO Kg-O_2_/kg-N	Organic Carbon Use kg-COD/kg-N
Traditional nitrification-denitrification	Autotrophic + heterotrophic	2–8	3.2	4.6	7.6
Shortened nitrification-denitrification	Autotrophic + heterotrophic	1.5	2.0	2.3	4.6
SHARON	Autotrophic + heterotrophic	1.5	1.0	2.3	2.4
OLAND	Autotrophic	0.1	0.16	1.7	0
ANAMMOX	Autotrophic	5.1	0.12	0	0
SHARON/ANAMMOX	Autotrophic	0.75	0.3	1.9	0
CANON	Autotrophic	1.2–8.9	0.3	2.1	0

NLR, nitrogen loading rate; DO, dissolved oxygen; COD, chemical oxygen demand; SHARON, stable high rate ammonium removal over nitrite; OLAND, oxygen limited autotrophic nitrification denitrification; CANON, completely autotrophic nitrogen removal over nitrite.

**Table 2:** Types of anammox process.

Processes	Types	Wastewater	Reactor Types	Reference
SHARON-Anammox	Two-Stage	Sludge digester effluents	-	[15]
CANON	One-Stage	Sludge digester effluents	SBR	[16]
DEMON	One-Stage	Sludge digester effluents	SBR	[17]
OLAND	One-Stage	Digested black water	RBC	[18]

SBR, sequencing batch reactor; RBC, rotating biological contactor.

**Table 3:** Comparison of the denitrification performance of CANON and OLAND processes.

Process	Wastewater Treated	Reactor Type	NLR kg-N/m^3^/d	NRR kg-N/m^3^/d
CANON	Synthetic	SBR	-	0.08
	Sludge digester effluents	SBR	0.46	0.36
	Sludge digester effluents	SBR	-	0.5
OLAND	Synthetic	SBR	0.13	0.05
	Digested black water	RBC	0.716	0.7

NRR, nitrogen removal rate.

**Table 4:** Application of anammox in technology to different types of wastewater.

Wastewater	Process	Scale	References
Piggery wastewater	HAOBR	Lab-scale	[39]
Nitrogen-rich saline wastewater	-	Lab-scale	[40]
Monosodium glutamate wastewater	PN/A (two-stage)	Lab-scale	[41]
Kitasamycin manufacturing wastewater	SBA-ANAMMOXb	Lab-scale	[42]
Landfill leachate	PN/A and Soil infiltration	Lab-scale	[43]
Domestic wastewater	CANON	Pilot-scale	[44]
Piggery wastewater	BNR	Full-scale	[45]

HAOBR, a new type of four-compartment hybrid anaerobic-aerobic baffle reactor; SBA, sequential biocatalyst addition; BNR, integrated fixed-film activated (IFA) sludge process.
